# Beyond symptoms: a multi-perspective study on youth with severe and enduring mental health problems

**DOI:** 10.3389/fpsyt.2025.1625102

**Published:** 2025-11-17

**Authors:** Chanel H. Bansema, Laura A. Nooteboom, Jacintha M. Tieskens, Lian Nijland, Rianne de Soet, Erik J. Giltay, Wouter G. Staal, Robert R. J. M. Vermeiren

**Affiliations:** 1 Department of Child and Adolescent Psychiatry, Leiden University Medical Center (LUMC) Curium, Leiden University Medical Center, Leiden, Netherlands; 2 Department of Psychiatry, Leiden University Medical Center, Leiden, Netherlands; 3 Karakter, Child and Adolescent Psychiatry, Nijmegen, Netherlands; 4 Department of Psychiatry, Radboud University Medical Center, Nijmegen, Netherlands; 5 Leiden Institution for Brain and Cognition, Leiden, Netherlands

**Keywords:** youth - young adults, severe mental health problems, characteristics, complexity, Likert scale questionnaire, multi perspectives, hopelessness

## Abstract

**Introduction:**

A group of youth in child-and-adolescent psychiatry (CAP) experiences severe and enduring mental health problems (SEMHP) transcending current classification systems. To support these youth timely and effectively, their characteristics require further exploration in clinical practice. Hence, this study aims to deepen our understanding of SEMHP characteristics in youth, taking into account perspectives from multiple stakeholders.

**Materials and methods:**

Following an exploratory sequential design identifying SEMHP characteristics initially in depth (in a literature and qualitative study), digital questionnaires were completed in three subgroups of in total 155 participants, 1) 81 youth (*Mage* = 21, *SD* = 3*)*, 2) 31 caregivers (*Mage* = 51, *SD* = 5*)*, and 3) 43 clinicians (*Mage =* 41, *SD* = 11), rating each characteristic. All participants described being familiar as youth with SEMHP, a caregiver of youth with SEMHP, or a clinician working with SEMHP, and thus able to evaluate their nature.

**Results:**

The characteristics prolonged suffering, several areas of life affected, interpersonal distrust, internalization of SEMHP, limited daily functioning, and hopelessness were consistently recognized by the three participant groups. Youth tend to score higher on the individual characteristics, with a significant difference between groups in the recognition of masking behavior. Family characteristics and unsafe environments are far less recognized by caregivers, while societal characteristics including societal ignorance, stigma and overemphasis on classifying are significantly less recognized by clinicians.

**Discussion:**

Youth, caregivers, and clinicians shared common ground in recognizing the pervasiveness of SEMHP. However, differences in perspectives on characteristics present challenges for diagnostics of these youth. Masking behavior of youth is unsurprising and indicates that these youth need a specific approach in diagnostics. A holistic and multi-perspective understanding of SEMHP is crucial for effective support, as care for these youth must take a systemic and connection-focused approach. Additionally, clinicians must be critically aware of the societal context.

## 1 Introduction

Child-and-adolescent psychiatry (CAP) services are struggling to provide adequate care for youth and young adults (referred to as ‘youth’ in this paper) who experience severe and enduring mental health problems (SEMHP) (1). The characteristics of this group seem to transcend the current categorical classification systems (2, 3), because of the multiplicity of mental health problems that are simultaneously expressed (4). For instance, youth with SEMHP frequently experience comorbid anxiety, depression, traumatic histories, emotional instability, profound hopelessness, suicide risk, self-destructive behavior, social distrust and impaired daily functioning (4). As a result, clinical practice in CAP considers these mental health problems to be “complex” or “challenging”, and experiences difficulties in meeting the needs of these youth (5). The impact of not (timely) recognizing such problems has significant implications for youth’s prognosis and current level of functioning (6). Therefore, improving recognition of SEMHP in youth is essential to alleviate the high burden of stress by these youth and their caregivers and clinicians (7). This requires a comprehensive understanding of the SEMHP characteristics and how these characteristics are related to youth, caregivers, and clinicians.

In understanding youth’s mental health problems, a developmental perspective is necessary (8). Youth with SEMHP, like all youth, are in a critical period in their life, namely adolescence, and disruptions in this period can have a serious impact on their well-being (9). While a developmental perspective is highly valued in psychiatry (10), more research is needed to increase knowledge about this period of life in the SEMHP group. This requires an approach considering interactions among biological, psychological and social factors, in multiple contexts of life (11). Two previous studies considered these crucial features in understanding SEMHP, by exploring biopsychosocial factors in SEMHP in the available existing literature (4) and the expression of SEMHP in clinical practice emphasizing the importance of including multiple contexts (4). The current study used these previous studies as a foundation to deepen insights into characteristics that contribute to the development and continuation of SEMHP.

First, the available literature suggests that youth with SEMHP experience severe functional impairments in academic, familiar, and social domains, and are burdened by prolonged stress, often resulting in suicidal ideation (4, 12, 13). Second, as experienced in clinical practice, these problems tend to be recurrent and persistent and are related to the duration of care (4, 14). Subsequently, youth often face long waiting lists for treatment, are rejected due to the complexity of their mental health problems or are misdiagnosed and consequently receive inadequate help (4, 15). Eventually, mental health problems that were not yet long-term eventually become severe and enduring. Despite much research into the importance of a holistic or ecological view in psychiatry (16), the context of the mental healthcare system itself remains relatively unexplored, especially for youth with SEMHP (4).

Moreover, it is notable that research on youth with SEMHP rarely incorporates a combined perspective from youth, caregivers, and clinicians. While previous studies have operated with questionnaires that were administered to the different groups (12), the level of agreement or how the perspectives align with one another were not examined. Both in research and clinical practice, alliance between youth, their caregivers and clinicians is important (17, 18). A prior study on youth showed that there was often no alliance regarding classified mental health problems in youth (14). A lack of alliance can result in disengagement in treatment (19) and potential drop-out of youth with SEMHP (5). Hence, it is essential to examine multiple perspectives which can strengthen alliance, improve diagnostics and treatment, and ultimately lead to more effective and supportive mental healthcare.

Following a multi-informant approach, the perspectives of youth, caregivers, and clinicians are fundamental (20, 21). Youth with SEMHP display mental health problems in specific contexts and not in others (treatment room versus home) (4). Therefore, in addition to youth’s unique perspectives on their experiences and needs (22), caregivers can provide insights into the characteristics of SEMHP in daily life and beyond the clinical setting (23). Lastly, the perspective of clinicians is needed to explore information on the manifestation of SEMHP in clinical practice and integrate their expertise to improve the quality of care provided to youth with SEMHP (24).

To deepen our understanding of SEMHP characteristics in clinical practice, an exploratory sequential design approach was followed (25). In prior research, we explored SEMHP characteristics by conducting qualitative approaches, including both a systematic review and a qualitative study (4). Based on these previous findings, the terms “severe” and “enduring” were described as many classified disorders, multiple mental health problems at once, prolong suffering, and long-term care histories. We found characteristics of SEMHP including, but not limited to, individual vulnerabilities such as heredity and (childhood) trauma, environmental factors such as parental psychiatry and lack of social support, mental health care factors such as overclassifying, societal invisibility and impaired functioning across life domains, and a sense of powerlessness among caregivers and clinicians. Hence, our current research questions will focus on (1) to what extent the characteristics of youth with SEMHP, as revealed by prior research, are recognized by youth with SEMHP, caregivers of youth with SEMHP, and clinicians working with youth with SEMHP, and (2) whether perspectives on the SEMHP characteristics differ between those stakeholders. Insight into similarities and differences can provide tools to engage in conversation with youth, caregivers, and clinicians during the diagnostic process.

## 2 Materials and methods

### 2.1 Study setting and design

This study is part of the ‘DevelopRoad’ project, centered on Dutch CAP facilities, with the goal of attaining a more profound understanding of the characteristics and needs of youth with SEMHP. The overall research project is explorative and follows an inductive grounded theory approach (4, 26). In this process, we continuously cycle through data collection, analysis, and reflection to explore characteristics of youth with SEMHP (4). Hence, this study is part of an exploratory sequential design. The first phase included a systematic review on the existing knowledge around SEMHP (4), and a qualitative study involving semi-structured interviews with youth with lived experience and specialized clinicians on the meaning and expression of SEMHP in clinical practice (4). The current study constitutes phase 2, aiming to examine whether these characteristics of youth with SEMHP are recognized by a larger sample of youth with SEMHP, caregivers of youth with SEMHP, and clinicians working with youth with SEMHP. The characteristics from phase 1 were translated into a Likert scale questionnaire which will guide the research process of this study (**Additional file 1**).

The DevelopRoad project team consisted of researchers, clinicians, and peer workers, associated with LUMC Curium, a CAP facility in the Netherlands. The Medical Ethics Review Board of Leiden University Medical Center concluded that the overall research project was not subject to the Medical Research Involving Human Subject Act (WMO) and complied with the Netherlands Code of Conduct for Research Integrity (reference number: N21.094).

### 2.2 Participants

Participants in this study consisted of three groups: (1) youth with SEMHP; (2) caregivers of youth with SEMHP; (3) clinicians working with youth with SEMHP. We described SEMHP as interrelated and enduring mental health problems that necessitate care, with often loss of all or part of youth’s hope for a better future (4, 5). To be included in this study, youth had to meet the following criteria: (a) aged 16–30 years; (b) are (or had been) in treatment in child-and-adolescent psychiatry (CAP); (c) because of SEMHP described as above. SEMHP was operationalized as self-identified severe and enduring mental health problems, in accordance with the description provided above, combined with current or past treatment in child-and-adolescent psychiatry. Participants were asked if they met the inclusion criteria as a form of verification. Those who indicated that they did not meet these criteria were unable to proceed with the questionnaire. Caregivers were included as main caregivers of youth with SEMHP, according to the description above. This could be both biological and nonbiological caregivers, however no information thereon was requested. Clinicians included, among others, psychiatrist, psychologist, and social workers with experience in working with youth with SEMHP in CAP. By including these participant groups, we explored characteristics in clinical practice, and gained insight into the degree of importance, relevance and potential differences between youth, caregivers, and clinicians. Based on prior research, we aimed to include a minimum of 30 participants in each group (27, 28). Due to the explorative character of this study a group of 30 participants seemed appropriate for an initial comparison. Participants were recruited using varied methods, for example by posting on social media, mailing (online) newsletters to clinicians in different CAP institutions (LUMC Curium, Levvel, Karakter, Accare, KieN GGZ), and approaching youth councils, expert-by-experience institutions (ExpEx and National Youth Council), and caregiver counsels. After the potential participants agreed to participate, they were asked to provide online informed consent before entering the questionnaire. A total of 155 participants were included. Informed consent was integrated into the questionnaire process, ensuring that without consent, participants could not proceed with the questionnaire. In addition, 57 gift vouchers of EUR 50, EUR 25, and EUR 10 were randomly awarded to participants.

### 2.3 Data collection and analysis

This study was performed with questionnaires using Castor EDC software (29). The questionnaires were validated by CB, RS, LAN and a LUMC specialist medical research data management. Data were collected between January and December 2022. The questionnaire consisted of six themes based on previous studies (4): (1) descriptions of the terms severe and enduring; (2) individual characteristics, divided into feelings and behavior; (3) family characteristics; (4) peer characteristics; (5) societal characteristics, including the mental healthcare setting; (6) the impact of experiencing SEMHP. Themes consisted of varying items associated with SEMHP (**Additional file 1**), and an open question providing the opportunity to list missing characteristics. No other characteristics emerged from the open ended-questions, only in-depth responses explaining the characteristics. In total 49 items were analyzed. Participants were asked to rate the extent to which they recognized the descriptions/characteristics of themselves (youth), their child (caregivers), or their clients with SEMHP (clinicians). The overall survey question was: To what extent does the characteristic below apply to your situation/your child’s situation/your client’s situation? For example *To what extent do you recognize: Often bullied or rejected by peers? or To what extent do you recognize: Wanting to numb yourself through self-harm?* Each characteristic was scored independently (not summed within the themes) for a possible score range of 1-5 (1 = Totally not, 2 = Hardly, 3= A bit, 4 = Mostly, 5= Totally). An additional option, 6= I do not know, was also available. The questions were not mandatory to complete, therefore it was possible to skip a characteristic. Questionnaires with at least 85% completed were included.

Characteristics were considered “unrecognized” when the mean of the response lied between 1 (totally not) and 2 (hardly) and considered “recognized” when the mean of the response lied between 4 (mostly) and 5 (totally) for all the groups. The internal consistency of the characteristics was acceptable, with a Cronbach’s alpha of.84. No formal validation analyses were conducted. To examine whether characteristics were differentially recognized by the three groups of participants (youth, caregiver, clinician) a one-way analysis of variance (ANOVA) was conducted for each characteristic, with group (youth, caregivers, clinicians) as the independent variable (30, 31). All the reported differences between participant groups remained significant after controlling the False Discovery Rate (FDR), a multiple significance testing approach by Benjamini and Hochberg (32). Following significant ANOVA results, we performed *post-hoc* pairwise comparisons using Tukey’s test to identify specific group differences. Adjusted p-values were reported, and comparisons with p <.05 were considered statistically significant. Effect sizes were estimated using omega squared (ω²). Effect sizes for omega squared (ω²) can be interpreted using Olejnik and Algina (33) guidelines, with small (ω² ≈ 0.01), medium (ω² ≈ 0.06), and large effects (ω² ≈ 0.14), indicating the proportion of variance explained by differences among the three groups. Skipped characteristics were not included in the analysis, and therefore group sizes may differ per characteristic. When a characteristic was recognized or unrecognized by one or two participant groups, but not by the other participant group(s), we classified it as “inconsistently recognized” or “inconsistently unrecognized”. Lastly, if a characteristic received varying mean scores and none of them were in the range of “recognized” or “unrecognized” we labelled it as “undetermined”. Additionally, we collected and analyzed demographic information on age, gender, educational level, and type of mental healthcare service to describe our sample (34). Computations and the visualization were done using R (version 4.3.2), with the package “forestplot” (version 3.1.3) (35).

## 3 Results

### 3.1 Demographics

A total of 155 participants completed the Likert scale questionnaire (youth *n* = 81, caregivers *n* = 31, and clinicians *n* = 43) (**Table 1**). Most participants were female (*n* = 132). Youth were 16–30 years old (*M* = 21, *SD* = 3), and most youth (85%) completed high school or further academics. Caregivers were 39–61 years old (*M* = 51, *SD* = 5), and just under half (45%) have completed university of applied sciences. Clinicians were 23–65 years old (*M* = 41, *SD* = 11), and most clinicians completed university of applied sciences (35%) and university (47%).

**Table 1:** Demographics of the participants (youth, caregivers and clinicians) are presented in percentages (%), except for age.

	Youth	Caregivers	Clinicians
Gender	%	%	%
Female Male Non-binary Do not want to share	85.2 4.9 7.4 2.5	93.5 6.5 0.0 0.0	79.1 20.9 0.0 0.0
Age (in years)	16-30	39-61	23-65
	(*M* = 21, *SD* = 3)	(*M* = 51, *SD* = 5)	(*M* = 41, *SD* = 11)
Completed highest education	%	%	%
Primary school High school MBO HBO WO	14.8 58.0 14.8 8.6 3.7	0.0 9.7 16.1 45.2 22.6	0.0 2.3 2.3 34.9 46.5
Duration of mental health problems	%	%	%
6–12 months 1–2 years 2–5 years 5–10 years >10 years “*I do not know”*	0.0 0.0 13.6 43.2 40.7 2.5	0.0 0.0 32.3 25.8 38.7 3.2	0.0 14.0 32.6 27.9 20.9 4.7
Type of additional received care for youth	%	%	%
District Team Ambulatory Youth Care Foster care Basic youth care Outpatient help Inpatient treatment in an institution Mentally handicapped assistance Supervision and guardianship Juvenile prison	30.9 56.8 8.6 75.3 76.5 82.7 0.0 7.4 1.2	0.0 3.2 0.0 6.5 9.7 29.0 0.0 6.5 0.0	0.0 0.0 0.0 0.0 4.7 34.9 0.0 0.0 0.0
Duration of provided care for youth	%	%	%
6–12 months 1–2 years 2–5 years 5–10 years >10 years “*I do not know”*	2.5 7.4 27.2 43.2 18.5 1.2	3.2 6.5 25.8 35.5 29.0 0.0	2.3 14.0 32.6 25.6 18.6 7.0

Caregivers and clinicians responded to questions regarding the duration of mental health problems, as well as the type and duration of care, in relation to their child or client, respectively.

### 3.2 Characteristics of the target group

**Figure 1:**
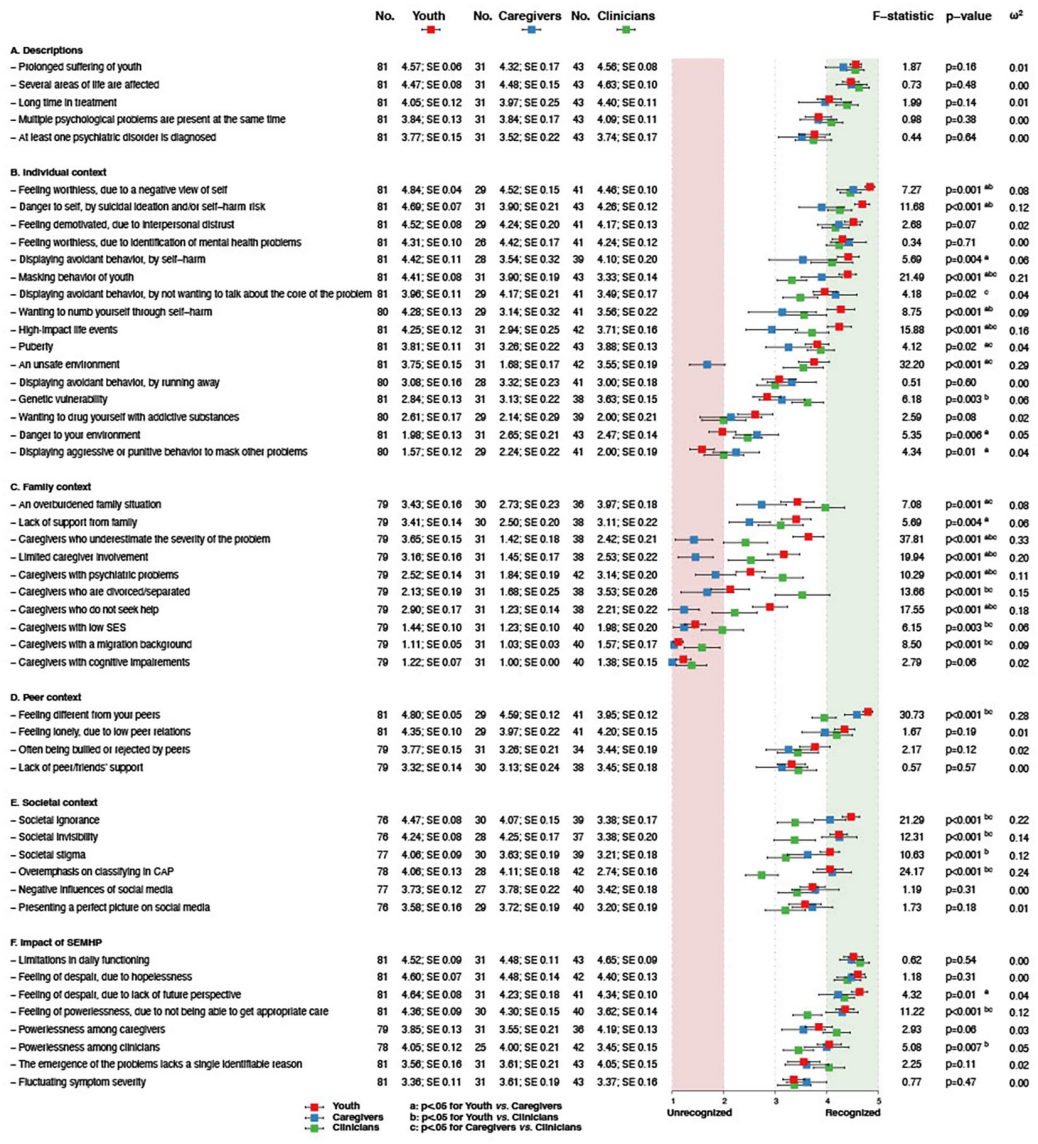
Forestplot of means of Likert scale responses by groups on SEMHP characteristics. Note: The forestplot represents the test statistics of separate one-way analysis of variance (ANOVA) for each characteristic to examine group differences (youth, caregiver, clinician). No. indicates the analytic sample, followed by the mean, and SE (standard error). ^a,b,c^ represents significant differences between the specific groups as indicated by the Tukey’s post-hoc test, where a=youth vs. caregivers, b=youth vs. clinicians, and c=caregivers vs. clinicians.

To increase understanding of SEMHP in youth, we examined to what extent the descriptions and characteristics were recognized by youth, caregivers, and clinicians (participant groups). **Figure 1** provides an overview with the means, standard errors, F-values, significancy levels and effect sizes, of the responses on the specific descriptions and characteristics for the participant groups. Four contexts were identified based on previous research (4), including: individual, family, peers, and societal context. In addition, we have focused on the impact of experiencing SEMHP. A summary of characteristic recognition status by participant group can be found in **Additional File 2**.

Participant groups consistently recognized the descriptions of severe as *several areas of life affected* and enduring as *prolonged suffering and long in treatment.*

In the individual context, participant groups consistently recognized *a negative view of self, interpersonal distrust*, and *identification of SEMHP*. However, significant inconsistencies were found concerning nine characteristics. Youth scored higher on 1) *masking*, 2) *self-harm to feel numb*, and 3) *high-impact life events*, compared to caregivers and clinicians. Both youth and clinicians scored higher on 4) *danger to self by self-harm* and 5) *avoidance by self-harm*, in contrast to caregivers. On the other hand, caregivers and youth scored higher on 6) *avoidance by not wanting to talk about the core of the problem*, in contrast to clinicians. In addition, youth and clinicians both scored lower on 7) *aggressive behavior to mask*, in contrast to caregivers. Moreover, youth scored lower than caregivers and clinicians on 8) *danger to environment*. Lastly, caregivers scored lower on 9) *unsafe home environment*, compared to youth and clinicians.

In the family context, participant groups consistently did not recognize *caregivers with cognitive impairments, a migration background*, and *a low socioeconomic status*, with significant differences between groups for the latter two characteristics. However, significant inconsistencies were found concerning six characteristics. Specifically, caregivers scored lower on *Caregivers who:* 1) *underestimate the severity of youth’s problems*, 2) *are limited involved*, 3) *with psychiatric problems*, 4) *are divorced/separated*, and 5) *do not seek help*, compared to youth and clinicians. Moreover, clinicians scored higher than youth and caregivers on 6) *an overburdened family situation*.

In the peer context, participant groups consistently recognized *feeling lonely, due to low peer relations* and *feeling different from peers*, with significant differences between groups for the latter characteristic.

In the societal context, no characteristics were consistently recognized by the participant groups. Significant inconsistencies were found concerning four characteristics. Youth and caregivers scored higher on 1) *societal ignorance*, 2) *societal invisibility*, and 3) *overemphasis on classifying in CAP*, compared to clinicians. Additionally, youth scored higher on 4) *societal stigma*, than caregivers and clinicians.

Regarding the impact of SEMHP on youth’s daily life, participant groups consistently recognized *limitations in daily functioning, feelings of despair in youth due to hopelessness*, and *feelings of despair in youth due to a lack of future perspective.* Significant inconsistencies were found concerning two characteristics, namely youth and caregivers scored higher on 1) *feelings of powerlessness due to not being able to get appropriate care* and 2) *powerlessness among clinicians*, compared to clinicians.

## 4 Discussion

In this study, we explored the characteristics of a group of youth who are rarely researched and about whom we know very little: youth with severe and enduring mental health problems (SEMHP). We examined SEMHP characteristics revealed by prior research (4), and have presented these to relevant stakeholders including youth, caregivers, and clinicians. While three participant groups shared common ground in the recognition of *prolonged suffering, several areas of life affected, interpersonal distrust, internalization of SEMHP, limitations in daily functioning*, and *hopelessness*, there were differences between perspectives on crucial characteristics. We identified significant differences on trauma-, caregiver- and societal related characteristics, as well on self-harm and whether youth show masking behavior. Significant differences between perspectives of the stakeholders may hinder timely and adequately recognition of SEMHP in clinical practice. With the combination of differences in perspectives on trauma, masking, societal invisibility and stigma, there is a chance that caregivers and clinicians largely overlook the impact, while youth feel acutely aware of it. As a result, youth find themselves trapped in a vicious cycle of unintentionally being overlooked and feeling invisible, as well as risk behavior such as suicidality and self-harm.

### 4.1 Consistent recognized characteristics of SEMHP

Multiple characteristics were consistently recognized among youth, caregivers, and clinicians including *prolonged suffering, interpersonal distrust, a negative view of self, internalization of SEMHP, feelings of loneliness and being different, hopelessness*, and *limited daily functioning.* These characteristics pose substantial risks to youth’s wellbeing and resilience. First, prior research showed that feeling alienated from peers contributes to a low sense of belonging (36) and is associated with detrimental consequences such as suicidality (37). This adds further risk for youth with SEMHP, intensifying an already existing tense for self-harming behavior. Second, these characteristics contrast with the traits required for resilient development. Resilience, defined by Masten et al. (38) as a multisystemic dynamic process of adapting to or recovering from adversity, depends on the interaction of individual, familial, social and broader ecological systems. A similar process shapes the course of SEMHP among youth. From this multisystemic perspective, resilience and recovery of youth with SEMHP involves not only addressing individual vulnerabilities (e.g., interpersonal distrust, self-harm), but also strengthening broader social resources such as family support, peer relations, and adequate mental health care.

### 4.2 Inconsistent recognized characteristics of SEMHP

Moreover, besides similarities in perspectives, we identified significant differences in perspectives between participant groups on SEMHP characteristics. These differences are highly relevant for clinical practice and should be discussed properly, as the connection between youth, caregivers, and clinicians is highly important.

First, in line with prior research (39, 40), we found significant differences in the recognition of trauma-related characteristics. Notably, caregivers did not recognize *an unsafe home environment*, in contrast to youth. This discrepancy may stem from caregivers trivializing their actions as discipline or valid punishment, rather than recognizing them as harmful (40–42). Similarly, caregivers did not recognize caregiver-related characteristics, such as *underestimation of severity* or *limited involvement.* Prior research showed that caregivers may misinterpret youth’s withdrawal as normative adolescent separation, rather than a manifestation of mental health problems (43, 44). Also, the evolving self-concept during adolescence may isolate youth’s perspective, further complicating mutual understanding (45). Second, our findings reveal a notable difference in perspectives on self-harm. Youth recognized *self-harm* as means *to avoid* or *to feel numb*, in contrast to caregivers. While recent research has been done on self-harm on adolescents (46), the difference in perception between youth and caregivers lacks exploration. Our study highlights the need for future research to increase insight into the underlying mechanisms of self-harm and to facilitate clinicians for a meaningful dialogue between youth and caregivers.

Moreover, youth uniquely recognized *masking behavior of youth*, contrasting caregivers and clinicians. While masking is explored in a few studies (47, 48), it lacks proper exploration for youth with SEMHP including multiple perspectives. Future research should further investigate this, as youth masking could help explain the perceptual differences identified in our study. For example, youth may mask their problems due to perceived burdensomeness (4), resulting in caregivers limited awareness of their experiences. Importantly, a resulting danger of these differences in perspectives may be an increase in perceived lack of social support and loneliness in youth (49).

Lastly, both youth and caregivers recognized *overemphasis on classifying in CAP* as part of SEMHP, a perspective not shared by most clinicians. Clinicians may (un)consciously rely on diagnostic labels following the traditional medical model which has been the core of their education and training, and out of necessity for resource allocation (50–52). However, reliance on diagnostic labels may interfere with understanding of SEMHP, as labels alone fail to capture contextual factors or the complexity of these problems (53, 54). Moreover, youth recognized societal characteristics such as *societal stigma*, whereas clinicians, likely due to their professional exposure, may overlook these problems. This is worrying, since societal stigma may worsen youth’s loneliness and view of the world (55, 56). Our findings emphasize the need for clinicians to critically examine their own perspectives and integrate an awareness of societal characteristics when assessing SEMHP in youth.

### 4.3 Implications

This study highlights the complexity of understanding youth with SEMHP, since their characteristics are recognized differently by youth, caregivers, and clinicians. In this section, we discuss implications for future research and clinical practice to improve the recognition of youth with SEMHP. We emphasize the need for a holistic approach, including multiple perspectives, in both studying youth with SEMHP and assessing their characteristics during diagnostics in clinical practice.

First, youth with SEMHP are frequently described as “complex” in child-and-adolescent psychiatry, though definitions of complexity vary, ranging from severity of impairment, to intensive service use and comorbid conditions (57). Consensus on what constitutes clinical or mental health complexity among youth remains absent. A holistic perspective is therefore essential, recognizing how co-occurring mental health problems, social stressors, and functional impairments interact to shape these difficulties (57, 58). Our study has explored these contributing factors from the perspectives of multiple stakeholders, with future research needed to examine their interactions in the development and continuation of SEMHP. Moreover, both youth’s and caregivers’ perspectives on SEMHP characteristics must be integrated properly into diagnostic procedures. While this seems as the obvious, our study identified clinically relevant differences on SEMHP characteristics, that seem not yet discussed in a proper manner. This specifically concerns trauma-related characteristics, caregiver-related characteristics, and youth’s masking behavior. Discussing these differences can enhance mutual understanding and increase perceived support (59, 60). Additionally, clinicians must be aware of their own perspectives as they differ from those of youth and caregivers, such as on *masking* and *societal stigma*, and *overemphasis on classifying.*

Furthermore, clinicians should be mindful of a potential tendency towards DSM-5 classifications (2), as it does not capture youth’s whole story. First, while it may capture characteristics such as *the impact on daily functioning* or *the view of self*, it also lacks a deeper understanding of the underlying problems (61, 62). For example, it fails to address masking behavior or reasons to self-harm. Second, although classification systems are often valued for enabling treatment standardization (63), they may also exclude youth with SEMHP whose needs do not fit within diagnostic categories (5, 61). Lastly, while classification systems can facilitate communication between professionals (63), it may not improve conversations with youth as it can lead to diagnostic alienation, where youth feel labelled rather than understood. Recognizing that existing frameworks for psychiatric classification and treatment seem insufficient for “complex” mental health problems such as SEMHP among youth, emerging approaches as transdiagnostic clinical staging models have gained prominence. These approaches emphasize early intervention and prevention through stage-specific, individualized care that also accounts for environmental factors (64). In addition, the International Classification of Functioning for Children and Youth (ICF-CY) (65) incorporates both personal and environmental factors considering youth’s development (65). These holistic approaches are essential for understanding SEMHP among youth (66–68). Yet, translating a holistic view into a comprehensive narrative in which youth can recognize themselves remains challenging. Emerging diagnostic approaches in the Netherlands, prioritizing personal narratives and a person-centered focus are among others the Patterns of Life project for adults (69) and a shared explanatory analysis (70). Further research could explore how these approaches impact diagnosis and treatment for youth with SEMHP.

### 4.4 Strengths and limitations

The groundwork for this study is based on the existing literature (4) and interviews from a previous study (4). The data of these prior studies are mainly collected by conducting qualitative methods, which is highly useful to gain in-depth information in small groups and in a particular context (71). However, the transferability of such data could be limited, because a relatively small group of youth do not represent the whole target group (72). Therefore, to increase the transferability of the characteristics, we decided to include a larger and multi-perspective group (73). This approach with this target group is to our knowledge the first and increases the validity and generalizability of our results.

A strength of this study is the inclusion of our target group: a new generation of youth. This generation faces specific societal problems (e.g., social media, and the experience of COVID-19) emphasizing the need to conduct and continue conducting research on these youth and their (new generation) needs (74). In this study, we used an age range of up to 30 years to reach a larger group of youth (including youth with a history of treatment in CAP), so that they could reflect on their prior experiences. Since there is little known about youth with SEMHP, including young adults could provide valuable information about transitions in life, such as identity development or societal innovations. However, generation experiences and needs may differ between youth aged 16 and those aged 30. Moreover, through the application of Likert scale questionnaires we were able to deepen our insight into the relevance of characteristics of youth with SEMHP and in similarities and differences between perspectives.

These insights have high clinical value. However, the results should be considered within the context of the following limitations. First, the distribution of participants across the three groups was uneven, with a scarcity of caregivers and clinicians. The challenges in recruitment of caregivers may be explained by caregivers’ concerns about privacy and stigma, or the perception that their input might not be as impactful as that of clinicians or youth themselves. Another potential explanation for the scarcity of caregivers and clinicians may be a lack of time and/or heavy workload (75). On the other hand, a relatively large number of youth participated in this study, confirming the importance for youth themselves to increase understanding of SEMHP from their unique perspectives. The imbalance in group sizes between youth and the two other participant groups may have influenced the group comparisons. It is possible that participation of more caregivers would yield more recognition of trauma-related characteristics; this is also true of societal-related characteristics among clinicians. Yet, it is notable that we identified significant differences between youth and one of the other participant groups on these characteristics, whereas the other group, despite its smaller size, showed more similarity to the youth participant group. Future research including a larger number of caregivers and clinicians could increase understanding of the differences in perspectives on these characteristics. Second, as this study is solely conducted in the Netherlands, generalizations of our results to other Western countries or non-Western countries may be limited. However, a recent study on other Western countries identified similar results concerning the impact of societal stressors on youth’s mental health (76). We also believe youth in non-Western countries are dealing with severe and enduring mental health problems, including suicidality, self-harm, family dysfunctioning and low-self-esteem, however potentially also more related to violence and poverty (77). Moreover, participants in this study consistently did not recognize *migration background* as a SEMHP characteristic, while previous research did identify this characteristic in relation to severe mental health problems in youth (78, 79). This difference indicates potential bias of our group composition. Also, the overrepresentation of females in all participant groups indicates a potential gender bias (80). Therefore, to address this imbalance it is essential for future research to explore male engagement and potential underrepresentation of migrant background families within the CAP setting (81, 82).

## 5 Conclusion

While youth, caregivers, and clinicians shared common ground in recognizing SEMHP characteristics as prolonged suffering, several affected life domains and hopelessness, there are differences between perspectives on crucial characteristics. We identified significant differences on trauma-, caregiver- and societal related characteristics, as well on self-harm and whether youth show masking behavior. These differences are clinically relevant, as they may contribute to misunderstanding and feeling unheard by all stakeholders. In all, this study adds to literature calling for a better understanding and recognition of youth with SEMHP, emphasizing the need for a holistic and multi-perspective approach to diagnostics. To continuously stay attuned on the perspectives and ensure that dynamics that often lead to SEMHP are addressed timely, diagnostics should span the entire duration of care. Future research on transdiagnostic approaches is needed to do justice to the underlying dynamics and highly contextual nature of SEMHP.

## Data Availability

The raw data supporting the conclusions of this article will be made available by the authors, without undue reservation.

## Supplementary material

Additional file 1 Likert scale questionnaire for youth, based on prior research. These questionnaires were adjusted for caregivers and clinicians. Bolded questions were utilized for this study, the other questions have been gathered for the overall DevelopRoad project.

Additional file 2 Summary of characteristic recognition status by participant group. *The Likert scale scores were rounded either up or down, depending on the decimal value.
