# Supplementary material for: Beyond symptoms: a multi-perspective study on youth with severe and enduring mental health problems
Source: Front Psychiatry. 2025 Nov 17;16:1625102. doi: 10.3389/fpsyt.2025.1625102 (PMC12667609; doi:10.3389/fpsyt.2025.1625102)
Supplement: Additional file 1 — Likert scale questionnaire for youth, based on prior research. These questionnaires were adjusted for caregivers and clinicians. Bolded questions were utilized for this study, the other questions have been gathered for the overall DevelopRoad project. [file DataSheet1.pdf]

Additional file 1. Likert scale questionnaire for youth, based on prior research

Category	Theme	Type of question	Characteristic
Descriptions of severe and enduring mental health problems	Enduring	**Likert scale**	**Prolonged suffering**
		**Likert scale**	**Duration of care**
		Open	
	Severe	**Likert scale**	**At least one psychiatric disorder**
		**Likert scale**	**Co-occurrence of mental health problems**
		**Likert scale**	**Several life domains affected**
		Open	
Characteristics of severe and enduring mental health problems		**Likert scale**	**High burden of suffering**
		**Likert scale**	**Limitations in daily functioning**
		Likert scale	Extreme feelings or behaviors
		**Likert scale**	**Danger to yourself in the form of suicidal thoughts and self-harm**
		**Likert scale**	**Danger to your environment e.g., aggressive behavior**
		**Likert scale**	**No single identifiable reason for the emergence of the problems**
		**Likert scale**	**Changing severity of the problems: the problems are present/limited to varying degrees**
		**Likert scale**	**The invisibility of the problem due to the prolonged display of socially desirable behavior (masking)**
		Open	
Type of mental health problems		Likert scale	Externalizing problems
		Likert scale	Internalizing problems
		Likert scale	Combination of externalizing and internalizing
		Likert scale	Social-emotional problems: disturbing thoughts, emotions, and behavior
		Likert scale	Suicidal thoughts
		Likert scale	Suicidal behavior
		Open	
Vulnerabilities		**Likert scale**	**Genetic vulnerability to develop a certain disorder: it runs in the family**
		**Likert scale**	**An unsafe environment, such as mistreatment, neglect or abuse (emotional or physical)**
		**Likert scale**	**Intense (life) events**
		**Likert scale**	**Puberty as an extra vulnerable period for developing psychological problems**
		Open	
Effects of mental health problems on youth	Feelings	**Likert scale**	**Feeling despair due to loss of hope for everyday life and being part of society**
		**Likert scale**	**Feeling of despair due to lack of future perspective**
		**Likert scale**	**Feeling of powerlessness by not being able to get appropriate care**
		Likert scale	Feeling of powerlessness by not experiencing direction in the care process
		**Likert scale**	**Feeling of worthlessness due to having a very negative self-image**

		**Likert scale**	**Feeling of worthlessness because you have come to see the problems as part of your identity**
		Likert scale	Feeling demotivated by a lack of trust in the mental healthcare service
		**Likert scale**	**Feeling demotivated by a lack of confidence in yourself (interpersonal distrust)**
		**Likert scale**	**The feeling of being different from your peers**
		**Likert scale**	**Feeling lonely due to low quality of social relationships**
	Behavior	**Likert scale**	**Displaying avoidant behavior in the form of self-harm**
		**Likert scale**	**Displaying avoidant behavior by not wanting to talk about the core of a problem**
		**Likert scale**	**Displaying avoidant behavior by running away**
		**Likert scale**	**Displaying aggressive or punitive behavior to mask other problems**
		**Likert scale**	**Wanting to numb yourself through addictive substances**
		**Likert scale**	**Wanting to numb yourself through self-harm**
		Open	
The environmental role	Caregivers/family	**Likert scale**	**Low socio-economic status, e.g., poverty and unemployment**
		**Likert scale**	**Caregivers with a migration background**
		**Likert scale**	**Caregivers with psychiatric problems**
		**Likert scale**	**Caregivers with (mild) mental disabilities**
		Likert scale	First or second-degree relatives with addictions
		**Likert scale**	**Caregivers who are divorced/no longer together**
		**Likert scale**	**Caregivers who are insufficiently involved**
		**Likert scale**	**Caregivers who underestimate the severity of the problem**
		**Likert scale**	**Caregivers who avoid care**
		**Likert scale**	**An overburdened family situation**
		**Likert scale**	**Caregivers who feel powerless**
	Social network	**Likert scale**	**Often bullied or rejected by peers**
		**Likert scale**	**Getting little to no support from friends**
		**Likert scale**	**Getting little to no support from family members**
		Likert scale	Lack of knowledge and expertise in schools to identify and deal with mental health problems
		Open	
The role of the mental healthcare system		Likert scale	Involvement of many care providers who do not work well together
		Likert scale	Long waiting times for appropriate (specialist) care
		**Likert scale**	**Focus on classifying and treating single problems, resulting in insufficient awareness of the connection between problems in CAP**
		Likert scale	Not being allowed to enter treatment with specific organizations due to contraindications
		Likert scale	A gap/inadequate connection between youth and adult care (18-/18+)
		**Likert scale**	**Powerlessness of treatment providers**
		Open	

The societal role		**Likert scale**	**Stigma/negative perception in society when it comes to mental health problems in general**
		**Likert scale**	**Ignorance in society about the impact of severe and enduring mental health problems on youth**
		**Likert scale**	**The invisibility of this group of youth in society results in them needing to be heard and involved more**
		**Likert scale**	**Negative influences of social media**
		**Likert scale**	**Presenting a perfect picture on social media**
		Open	

These questionnaires were adjusted for caregivers and clinicians. Bolded questions were utilized for this study, the other questions have been gathered for the overall DevelopRoad project.
