# Supplementary material for: Beyond symptoms: a multi-perspective study on youth with severe and enduring mental health problems
Source: Front Psychiatry. 2025 Nov 17;16:1625102. doi: 10.3389/fpsyt.2025.1625102 (PMC12667609; doi:10.3389/fpsyt.2025.1625102)
Supplement: Additional file 2 — Summary of characteristic recognition status by participant group. *The Likert scale scores were rounded either up or down, depending on the decimal value. [file DataSheet2.pdf]

Additional file 2. Summary of characteristic recognition status by participant group

Characteristics	Participant groups			
**Descriptions**	**Youth**	**Caregivers**	**Clinicians**	**Combined**
Prolonged suffering of youth	Recognized	Recognized	Recognized	Consistent
Several areas of life are affected	Recognized	Recognized	Recognized	Consistent
Long time in treatment	Recognized	Recognized*	Recognized	Consistent
Multiple psychological problems are present at the same time	Undetermined	Undetermined	Recognized	Not significant
At least one psychiatric disorder is diagnosed	Undetermined	Undetermined	Undetermined	
**Individual context**				
Feeling worthless, due to a negative view of self	Recognized	Recognized	Recognized	Consistent
Danger to self, by suicidal ideation and/or self-harm risk	Recognized	Undetermined	Recognized	Inconsistent
Feeling demotivated, due to interpersonal distrust	Recognized	Recognized	Recognized	Consistent
Feeling worthless, due to identification of mental health problems	Recognized	Recognized	Recognized	Consistent
Displaying avoidant behavior, by self-harm	Recognized	Undetermined	Recognized	Inconsistent
Masking behavior of youth	Recognized	Undetermined	Undetermined	Inconsistent
Displaying avoidant behavior, by not wanting to talk about the core of the problem	Recognized*	Recognized	Undetermined	Inconsistent
Wanting to numb yourself through self-harm	Recognized	Undetermined	Undetermined	Inconsistent
High-impact life events	Recognized	Undetermined	Undetermined	Inconsistent
Puberty	Undetermined	Undetermined	Undetermined	
Unsafe home environment	Undetermined	Unrecognized	Undetermined	Inconsistent
Displaying avoidant behavior, by running away	Undetermined	Undetermined	Undetermined	
Genetic vulnerability	Undetermined	Undetermined	Undetermined	
Wanting to drug yourself with addictive substances	Undetermined	Undetermined	Unrecognized	Not significant
Danger to your environment	Unrecognized*	Undetermined	Undetermined	Inconsistent
Displaying aggressive or punitive behavior to mask other problems	Unrecognized	Undetermined	Unrecognized	Inconsistent
**Family context**				
An overburdened family situation	Undetermined	Undetermined	Recognized*	Inconsistent
Lack of social support from family	Undetermined	Undetermined	Undetermined	
Caregivers who underestimate the severity of problems	Undetermined	Unrecognized	Undetermined	Inconsistent
Limited caregiver involvement	Undetermined	Unrecognized	Undetermined	Inconsistent
Caregivers with psychiatric problems	Undetermined	Unrecognized	Undetermined	Inconsistent
Caregivers who are divorced/separated	Undetermined	Unrecognized	Undetermined	Inconsistent
Caregivers who do not seek help	Undetermined	Unrecognized	Undetermined	Inconsistent
Caregivers with low SES	Unrecognized	Unrecognized	Unrecognized*	Consistent
Caregivers with a migration background	Unrecognized	Unrecognized	Unrecognized	Consistent
Caregivers with cognitive impairments	Unrecognized	Unrecognized	Unrecognized	Consistent
**Peer context**				
Feeling different from your peers	Recognized	Recognized	Recognized*	Consistent
Feeling lonely, due to low peer relations	Recognized	Recognized*	Recognized	Consistent
Often being bullied or rejected by peers	Undetermined	Undetermined	Undetermined	
Lack of peer/friends' support	Undetermined	Undetermined	Undetermined	
**Societal context**				

Societal ignorance	Recognized	Recognized	Undetermined	Inconsistent
Societal invisibility	Recognized	Recognized	Undetermined	Inconsistent
Societal stigma	Recognized	Undetermined	Undetermined	Inconsistent
Overemphasis on classifying in CAP	Recognized	Recognized	Undetermined	Inconsistent
Negative influences of social media	Undetermined	Undetermined	Undetermined	
Presenting a perfect picture on social media	Undetermined	Undetermined	Undetermined	
**Impact of SEMHP**				
Limitations in daily functioning	Recognized	Recognized	Recognized	Consistent
Feeling of despair, due to hopelessness	Recognized	Recognized	Recognized	Consistent
Feeling of despair, due to lack of future perspective	Recognized	Recognized	Recognized	Consistent
Feeling of powerlessness, due to not being able to get appropriate care	Recognized	Recognized	Undetermined	Inconsistent
Powerlessness among caregivers	Undetermined	Undetermined	Recognized	Not significant
Powerlessness among clinicians	Recognized	Recognized	Undetermined	Inconsistent
The emergence of the problems lacks a single identifiable reason	Undetermined	Undetermined	Recognized	Not significant
Fluctuating symptom severity	Undetermined	Undetermined	Undetermined	

*The Likert scale scores were rounded either up or down, depending on the decimal value.
